# Telbivudine treatment started in early and middle pregnancy completely blocks HBV vertical transmission

**DOI:** 10.1186/s12876-017-0608-7

**Published:** 2017-04-13

**Authors:** Weihui Sun, Shangfei Zhao, Lei Ma, Anhua Hao, Bo Zhao, Lin Zhou, Fengzhu Li, Mingquan Song

**Affiliations:** 1 Department of Hepatology, Chengyang People’s Hospital of Qingdao, Qingdao, 266109 Shandong Province China; 2 Department of Gastroenterology, The Affiliated Hospital of Qingdao University, Qingdao, 266011 Shandong Province China

**Keywords:** Telbivudine, Hepatitis B virus, Mother-to-infant transmission, Fetus safety, Drug efficacy

## Abstract

**Background:**

To evaluate the efficacy and safety of treating HBV-positive mothers with telbivudine in early and middle pregnancy to prevent mother-to-infant HBV transmission.

**Methods:**

The subject population comprised pregnant women with chronic hepatitis B (CHB; *n* = 188) from January 2013 to June 2015, with HBV DNA ≥1.0 × 10^7^copies/mL and increased alanine aminotransferase levels. Groups A (*n* = 62) and B (*n* = 61) were treated with telbivudine starting at 12 weeks or 20–28 weeks after gestation, respectively. Telbivudine was discontinued at postpartum 12 weeks. Group C (*n* = 65) received no antiviral. All infants were vaccinated with hepatitis B immunoglobulin (200 IU) and HBV vaccine (20 with hepatitis B The maternal HBV DNA levels of the groups were compared. Mother-to-infant transmission of HBV was indicated by the presence of HBsAg in infants 7 months after birth.

**Results:**

Before treatment, the HBV DNA levels of the 3 groups were similar. Before delivery and 12 weeks after delivery, the HBV DNA levels of groups A and B were similar, but both were significantly lower than that of group C (*P* < 0.01, all). No infants in groups A and B were HBsAg-positive, but the infection rate of group C was 18.4% (*P* < 0.01). The HBV infection rate of infants was positively associated with the HBV DNA levels of the pregnant mothers.

**Conclusion:**

Administration of telbivudine to HBV-infected mothers, started during early and middle pregnancy, completely blocked mother-to-infant HBV transmission.

**Trial registration:**

The study was registered retrospectively on Janurary 25 in 2016 at Chinese Clinical Trial Registry (ChiCTR-OPC-16007899).

## Background

Chronic hepatitis B virus (HBV) infection is a serious health problem in many parts of the world. Persistent HBV infection can cause chronic liver injury that may advance to cirrhosis and hepatocellular carcinoma [1].

In regions where HBV infection is endemic, the main transmission route is mother to infant, and may be intrauterine, perinatal, or lactational [2]. Although passive and active immunoprophylaxis is widely implemented at birth (i.e., hepatitis B immunoglobulin and HBC vaccine), HBV transmission still occurs in 5% to 10% of immunized infants [3]. A risk factor for immunization failure is high maternal HBV load [4]. Therefore, antiviral therapy to reduce maternal HBV DNA levels during pregnancy has been employed to block mother-to-infant transmission.

Telbivudine is a synthetic thymidine nucleoside analogue that is approved as a pregnancy category B medicine for treating chronic hepatitis B [4, 5]. Many studies have reported that telbivudine administrated during the second and third trimester of pregnancy is highly safe for effectively interrupting mother-to-infant HBV transmission [6, 7]. However, the efficacy and safety of telbivudine treatment in early pregnancy has not been adequately investigated.

To identify effective approaches for preventing mother-to-infant transmission of HBV, the present study evaluated the efficacy and safety of telbivudine, begun in early and middle pregnancy, for treating HBV-positive pregnant women.

## Methods

### Patients

The Institutional Review Board of Qingdao Municipal Hospital had approved the study. Pregnant women (*n* = 188) at Chengyang People’s Hospital of Qingdao, China from January 2013 to June 2015 who met the following inclusion criteria were enrolled on a voluntary basis: 20 to 35 years old; 12 weeks or 20–28 weeks gestation; HBsAg- and HBeAg-positive, and HBV DNA ≥ 1.0 × 10^7^ copies/mL; elevated alanine aminotransferase (ALT), 40 to 400 U/L.

Criteria for exclusion were: received previous treatment with interferon or nucleos(t)ide analogs, coinfection with hepatitis A, C, D, or E virus, human immunodeficiency virus, rubella virus, cytomegalovirus, or simple herpes virus, syphilis, or *Toxoplasma gondii*; other antiviral drug medication, immunosuppressive or cytotoxic drugs, or corticosteroids during pregnancy; fetal deformity indicated by ultrasound examination; genetic disease; gestational diabetes or severe respiratory, urinary, or neurological comorbidity or severe gastrointestinal diseases which may affect drug absorption.

### Grouping and management

The women were allocated to 3 groups, A (*n* = 62), B (*n* = 61), and C (*n* = 65). In groups A and B, the women were at 12 weeks and 20 to 28 weeks of gestation, respectively; and all were given oral telbivudine 600 mg daily beginning immediately after the enrollment until week 12 postpartum. Group C received no antiviral therapy. All the women received glycyrrhizin to protect the liver. Maternal blood samples were collected before treatment, prior to delivery, and at postpartum 12 weeks to detect HBV DNA and ALT levels.

All infants born to the enrolled women were vaccinated with 200 IU of hepatitis B immunoglobulin within 6 h and at 30 days after birth, and 20 μg of hepatitis B vaccine within 12 h after birth, and at postpartum 4 and 24 weeks. The following infant data was collected at birth: height, weight, chest, and head circumference, 1-min Apgar score, and birth defects (the presence of any deformities). In addition, adverse pregnancy events (e.g., eclampsia, premature rupture of membranes, and premature delivery), postpartum hemorrhage, and cesarean section were recorded. Serum HBV DNA and HBsAg in infants were tested at week 28 postpartum.

### Statistical analyses

The data were analyzed using software SPSS 17.0. The chi-squared test was applied to compare categorical variables among groups. Continuous variables are presented as mean ± standard deviation. Differences among groups were compared with ANOVA test. *P* < 0.05 was considered statistically significant. The statistical analyses were performed by Li-Ping Su, Department Of Education, Chengyang People’s Hospital of Qingdao.

## Results

### Pregnancy characteristics

Groups A, B, and C were statistically similar with regard to age, infection duration, gravidity, delivery time, and gestational age at delivery (Table 1).

**Table 1:** Pregnancy characteristics and safety rates of the 3 groups

	Group A	Group B	Group C	P
Subjects, n	62	61	65	-
Age, y	28.9 ± 11.8	29.7 ± 9.8	27.5 ± 12.9	0.559
Infection duration, y	15.3 ± 10.9	16.1 ± 9.9	15.7 ± 11.5	0.919
Gravidity, n	1.8 ± 1.2	1.8 ± 1.1	1.8 ± 1.3	0.369
Delivery, n	1.1 ± 0.2	1.1 ± 0.1	1.1 ± 0.4	0.369
Gestational age, wk	39.0 ± 1.0	39.3 ± 1.2	39.2 ± 1.1	0.308
Adverse pregnancy, %	12.9(8/62)	11.4(7/61)	13.8(9/65)	0.923
Postparum hemorrhage, %	17.7(11/62)	14.7(9/61)	18.4(12/65)	0.844
Cesarean section, %	19.3(12/62)	16.4(10/61)	20.0(13/65)	0.859

### Serum HBV DNA levels before and after treatment

Before treatment with telbivudine, the serum HBV DNA levels of the 3 groups were statistically similar (Table 2). Before delivery, the serum HBV DNA levels of groups A and B were significantly lower than that of group C (*P* < 0.01, both). At week 12 postpartum, the differences in HBV DNA levels between groups A and C, and between groups B and C, were significantly different (*P* < 0.01; *P* < 0.01). There were no significant differences in HBV DNA levels between groups A and B before delivery or at 12 weeks postpartum (*P* = 0.934 and *P* = 0.923, respectively).

**Table 2:** Average HBV DNA and ALT levels before and after treatment

	Group A	Group B	Group C	P
Sujects, n	62	61	65	
HBV DNA viral load, log_10_ copies/ml				
Before treatment	7.79 ± 0.22	7.75 ± 0.19	7.74 ± 0.22	0.373
Before delivery	0.51 ± 1.44	0.49 ± 1.26	7.64 ± 0.24	<0.001
Postpartum 12 weeks	0.38 ± 1.12	0.35 ± 1.28	7.85 ± 0.27	<0.001
ALT levels, IU/mL				
Before treatment	125.3 ± 57.6	132.3 ± 52.9	128.5 ± 48.7	0.766
Before delivery	63.8 ± 15.6	59.5 ± 13.4	112.7 ± 24.1	<0.001
Postpartum 12 weeks	48.9 ± 18.2	51.3 ± 20.1	84.1 ± 32.5	<0.001

### ALT levels

Before treatment with telbivudine, there were no significant differences in ALT levels among groups A, B, and C (*P* > 0.05; Table 2). The differences between A and C, and between B and C were statistically significant before delivery (*P* < 0.01; *P* < 0.01), as well at week 12 postpartum (*P* < 0.01; *P* < 0.01). There were no significant differences in ALT levels between groups A and B before delivery or at postpartum 12 weeks (*P* = 0.104 and *P* = 0.488, respectively).

### Infant HBV infection rate at week 28 postpartum

The HBsAg positivity rates of infants in groups A, B, C at postpartum 28 weeks was 0%, 0%, and 18.4%, respectively (Table 3). The differences in HBV infection rate between groups A and C and between groups B and C were statistically significant (*P* < 0.01 and *P* < 0.01, respectively). The HBV DNA level of infants with HBsAg(+) was 6.04 ± 0.15 log copies/mL.

**Table 3:** General conditions and HBV infection of infants in the 3 groups

	Group A	Group B	Group C	P
Sujects, n	62	61	65	
Gestational age, wk	39.1 ± 1.1	39.2 ± 1.2	39.0 ± 1.1	0.613
Weight, g	3465.78 ± 408.5	3475.4 ± 398.4	3468.6 ± 401.3	0.991
Length, cm	50.4 ± 1.1	50.5 ± 1.3	50.6 ± 1.4	0.677
Head circumference, cm	33.5 ± 1.2	33.7 ± 1.1	33.3 ± 1.3	0.179
Apgar score	9.95 ± 0.2	9.97 ± 0.17	9.98 ± 0.18	0.648
HBV infection, n(%)	0	0	12(18.4)	<0.001

### Safety profile of telbivudine administrated during pregnancy

There were no significant differences in neonatal gestational age, body weight, height, head circumference, or Apgar score among the infants born to mothers in the 3 groups (Table 3). Among the 3 groups, there were no significant differences in the incidence of adverse pregnancy events (including eclampsia, premature rupture of membranes, premature birth), or in the rates of postpartum hemorrhage or cesarean section (Table 1). Among the patients enrolled, there were no virologic resistance found assessed by evaluating genotypic changes using HBV polymerase/reverse transcriptase assay, with absence of genotypic resistance in the telbivudine treatment.

## Discussion

In this study, we treated HBeAg-positive pregnant women with telbivudine beginning at 12 weeks or 20–28 weeks gestation until 28 weeks postpartum. All infants born to telbivudine-treated mothers were HBV-infection free, while the HBV infection rate of infants born to untreated mothers was 18.4%. Early treatment of HBV-positive pregnant women with telbivudine was effective and safe.

Chronic HBV infection in the Chinese general population is ~7.2% [8], and a significant portion of HBV-infected individuals are fertile women. The main route of HBV transmission is from mother to infant, and can occur during pregnancy or lactation. Combined passive and active immunization is unable to block completely this vertical HBV transmission. The reported mother-to-infant transmission rate can be as high as 15% [4], and was 18.4% in untreated pregnant women in the present study. This is consistent with the latest paper published in *N Engl J Med* by Pan et al. [9], the mother-to-infant transmission rate of the control group is 18%. The high mother-to-infant transmission rate was positively associated with serum HBV DNA levels in pregnant women prior to delivery [10]. This highlights the challenge of completely protecting infants from HBV infection.

Intrauterine transmission is an important cause for immunization failure, but the mechanisms for intrauterine infection are unclear. Viral, placental, and genetic factors, as well peripheral mononuclear cells are likely involved in intrauterine transmission. In early pregnancy, HBV can directly infect the fetus because the placental barrier has not formed. Even after formation of the placenta, abnormal pregnancy events such as maternal systemic toxemia can damage the placental barrier, leading to intrauterine HBV infection in early pregnancy [11].

As reported by Zou et al. [10], the HBV mother-to-infant transmission rate was positively associated with serum HBV DNA levels in pregnant women prior to delivery. If the HBV DNA load was effectively reduced during pregnancy, the intrauterine infection rate decreased [10]. In addition, pregnant women with active HBV replication may experience a flare-up of liver injury that can endanger the lives of both mother and fetus [12]. These circumstances support the antiviral treatment of HBV-positive women. We reasoned that earlier treatment may be more effective for blocking intrauterine HBV infection.

Telbivudine is an approved L-nucleoside drug for treating chronic HBV infection. Structurally, it is similar to lamivudine. Several studies have reported that telbivudine can effectively block mother-to-infant transmission in late pregnancy, with good tolerance and safety [6, 7, 13]. However, the efficacy and safety of telbivudine in treating HBV-positive women during early pregnancy has not been established. To our knowledge, our present study is the first report concerning early treatment of pregnant women with telbivudine. Consistent with our previous study [14], administration of telbivudine during early and middle pregnancy can reduce the rate of mother-to-infant transmission. Telbivudine treatment was also associated with normalized ALT, once HBV replication was at a low level, and an improved liver function fully supports successful pregnancy and delivery.

We found that the HBV DNA load of pregnant women prior to delivery and the HBsAg-positive rate of infants at 28 weeks postpartum were positively associated. In our study, the rate of vertical transmission in groups A and B was 0%, suggesting that telbivudine treatment was 100% effective in blocking HBV infection. There was no difference in the efficacy of telbivudine between groups A and B. This indicates that telbivudine treatment begun during middle pregnancy can be as effective as that started during early pregnancy. Because of the relatively small sample size in this study, our findings need to be verified in large cohorts.

Telbivudine is a pregnancy category B medication, and in preclinical studies showed no significant carcinogenic or teratogenic effects, nor organ, mitochondrial, or embryo-fetal toxicity. Furthermore, telbivudine inhibits viral DNA replication without affecting cellular DNA synthesis [15–17]. Some studies recommend initiating oral nucleoside analog therapy during the second or third trimester of pregnancy [18, 19]. Development of the vital organs of a fetus is essentially completed within the first 15 to 16 weeks of gestation [6]. Many studies have reported a safe profile for telbivudine used in the second and third trimester of pregnancy [6, 7]. Liu et al. [20] found that telbivudine used throughout pregnancy did not increase congenital malformations, indicated that its use was safe during early pregnancy. In the present study, we found no congenital deformity, severe adverse events, or complications in infants born to mothers who received telbivudine during early and middle pregnancy. Our findings further support the safe profile of telbivudine for pregnant women and their fetuses and infants.

The experience of drug related viral resistance is an important incident in anti-HBV treatment. Study have recognized that resistance may result in primary non-response or virologic breakthrough on therapy [21]. In addition, Nucleos(tide) analogs-related HBV resistance is associated with prior treatment with Nucleos(tide) analogs or treatment-naïve patients with high viral load [22]. A genotypic resistance test was performed in our included patients and data indicated that no patients enrolled developed with viral resistance. this may be because the limited period of antiviral treatment. All the patients stop antiviral treatment at 12 weeks after delivery. Study had reported that there were about 5% patients developing telbivudine related viral resistance during first year of treatment [23]. Therefore, whether or not to continue telbivudine treatment after delivery requires careful consideration.. Tenofovir is another pregnancy category B medication with high barrier of resistance [23]. Change from telbivudine with tenofovir after delivery if patients need prolong antiviral treatment may be a good clinical decision. However, there are still lack of evidence of safety data of tenofovir during lactation. Lamivudine is a well-tolerated anti-HBV Nucleoside analogues. It is effective in HBV suppression, ALT normalization and histologic improvement. However, viral breakthrough and drug resistance may occur after a long time LAM therapy with rtM204I/V ± rtL180M [24, 25]. HBV with substitutions rtM204I/V ± rtL180M has cross-resistance to telbivudine [21]. Patients with prior lamivudine exposure may not suitable use telbivudine treatment to blocks HBV vertical transmission. Although in our study, all patients included were treatment-naïve patients without potential lamivudine exposure and no patients found that experienced with telbivudine resistance during pregnancy. It still need to be concern the effective intervention to blocks HBV vertical transmission among CHB patients with potential prior lamivudine exposure or development of telbivudine resistance mutations during pregnancy. Tenofovir, the pregnancy category B nucleotide analogue that effective for both HBV and HIV, has shown to have superior HBV DNA suppression. Tenofovir is highly effective in the treatment of patients with rtM204I/V ± rtL180M HBV substitutions [21]. For blocks HBV vertical transmission among CHB patients with potential prior lamivudine exposure or development of telbivudine resistance mutations during pregnancy, initial tenofovir treatment or switch to tenofovir after telbivudine resistance may be a proper medical intervention. However, more clinical evidences are needed.

This study has a few limitations. Firstly, the sample size was relatively small, with only 188 subjects. Secondly, it was a single-center study, which requires verification in multi-center based investigations. Thirdly, we only studied the short-term safety of telbivudine for infants and a long-term follow-up is needed.

## Conclusions

Telbivudine administrated during early and middle pregnancy can completely block HBV mother-to-infant transmission. Early telbivudine treatment is safe for both pregnant mothers and their infants up to at least 28 weeks postpartum.

## Abbreviations

ALT: Alanine aminotransferase
CHB: Chronic hepatitis B
HBV: Hepatitis B virus
